# Early clear cell “sugar” lung cancer management: A case report and a brief literature review

**DOI:** 10.1111/1759-7714.13077

**Published:** 2019-04-16

**Authors:** Marsela Sjniari, Evelina Miele, Valeria Stati, Claudio Di Cristofano, Daniele Diso, Ylenia Pecoraro, Federico Venuta, Silverio Tomao, Gian Paolo Spinelli

**Affiliations:** ^1^ UOC of Oncology University of Rome Aprilia Italy; ^2^ Department of Hematology/Oncology and Stem Cell Transplantation Bambino Gesù Children's Hospital, IRCCS Rome Italy; ^3^ UOC of Pathology, Department of Medical‐Surgical Sciences and Bio‐Technologies University of Rome, Polo Pontino, I.C.O.T Latina Italy; ^4^ Department of Thoracic Surgery University of Rome Rome Italy

**Keywords:** Clear cell tumor, lung cancer, PEComa, sugar cancer

## Abstract

A clear cell tumor is a histological entity that rarely originates outside of the kidney. We describe a rare case of a clear cell tumor of the lung, also known as “sugar cancer,” that occurred in a 74 year‐old male patient, and perform a brief literature review. This report highlights the importance of an adequate disease management team, including surgeons, oncologists, and pathologists, to identify the best therapeutic approach to improve survival rates and the quality of life of patients affected by this rare disease.

## Introduction

Clear cell tumor of the lung (CCTL) is a rare histological subtype of large cell carcinoma according to the World Health Organization (WHO) classification, and is also known as a “sugar tumor” because there is an abundance of glycogen in the cytoplasm.^1^ The first case presentation was reported by Liebow and Castleman in 1963,^2^, ^3^ and since then approximately 60 cases have been reported in the literature.^4^

CCTL is typically observed in elderly,^5^ with no gender discrimination. The clinical presentation of this tumor is often associated with an asymptomatic pulmonary “coin lesion” on chest radiogram.^6^ Unspecific symptoms, such as fever, chest pain, breathlessness, cough, sense of suppression, and hemoptysis can lead to an incidental diagnosis of CCTL.^6^

These tumors belong to the family of perivascular epithelioid cell tumors (PECs).^7^ One of the main pathological findings of clear cell tumors is the large amount of cytoplasmic periodic acid‐Schiff (PAS)‐positive glycogen, with associated immunoreactivity for S‐100 protein and human melanoma black (HMB)‐45, while the absence of CK reactivity usually establishes the diagnosis.^8^ These immunohistochemical features discriminate CCTL from pulmonary metastasis related to primary renal cell carcinoma, clear cell variants of squamous cell carcinoma, and clear cell adenocarcinoma.^9^ Radiological features on contrast‐enhanced computed tomography (CT) show intense heterogeneous enhancement in the arterial phase (wash‐in) and washout in the delayed phase, as a probable result of the tumor vascular stroma.^10^, ^11^ Because of its low metastatic potential, CCTL generally has a benign prognosis, although a few malignant clinical cases have been reported.^12^, ^13^ More aggressive behavior has been associated with a larger diameter (> 2.5 cm), abundant mitoses, necrosis, and the presence of uncommon symptoms.^4^

Surgical resection has been demonstrated to be curative in CCTL, while the role of chemotherapy remains questionable.^4^

## Case report

**Figure 1:**
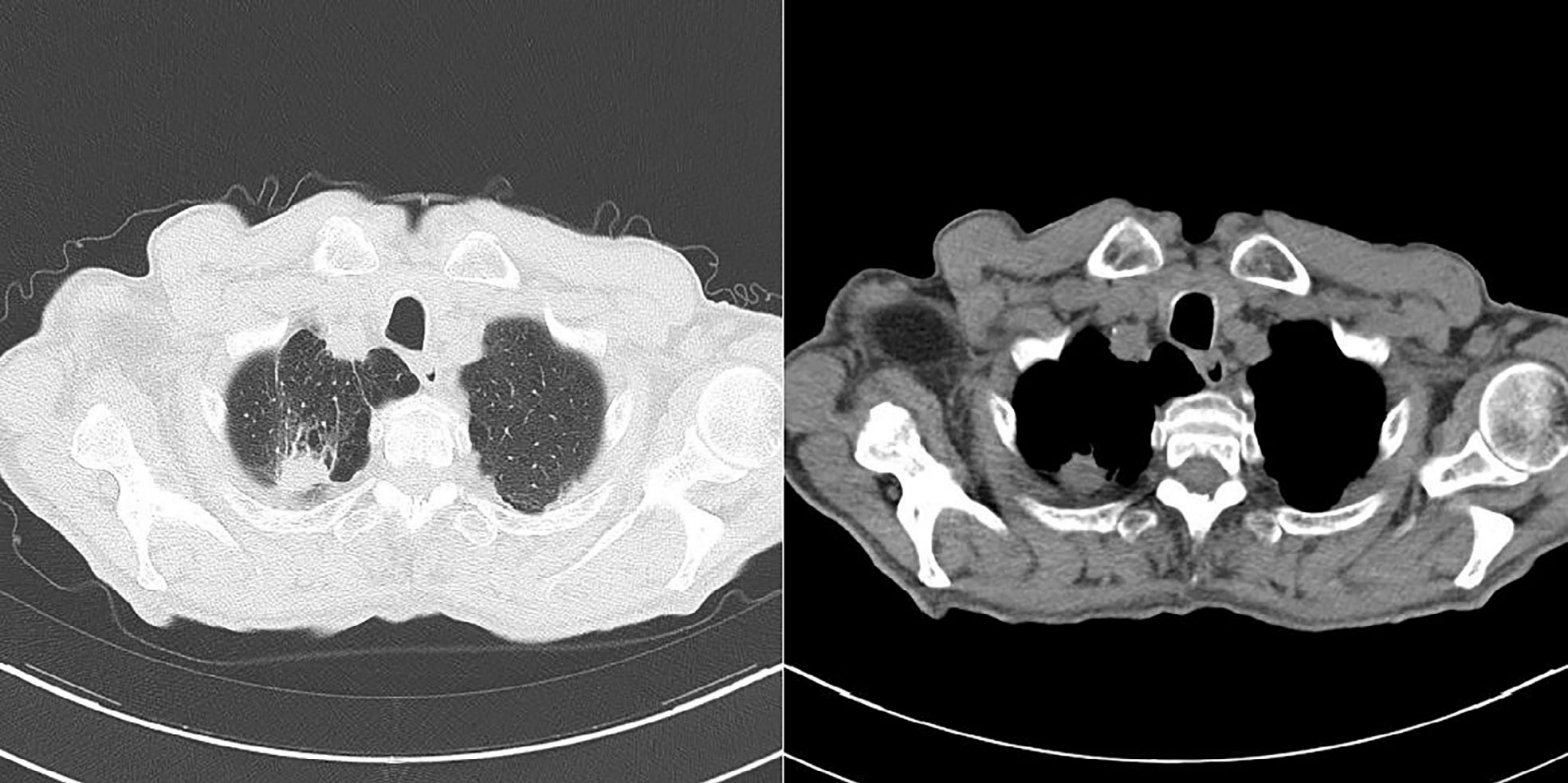
Subpleural lesion of the right lung, surrounded by bubbles of emphysema.

We present a case of 74‐year‐old man with a 22 mm subpleural pulmonary lesion in the apical portion of the right lung, detected by a CT scan performed during a pulmonary consult for his chronic obstructive pulmonary disease (COPD). Bubbles of emphysema surrounded the lesion, which is non‐specific for a diagnosis of CCTL (Fig 1).

To determine the histological nature, we performed a CT‐guided core biopsy of the lesion. Microscopic examination revealed a clear cell non‐small cell lung carcinoma with focal and weak nuclear positivity to TTF‐1 and negativity to p63 and synaptophysin reactivity. A staging total body contrast‐enhanced CT scan revealed a 28 mm lesion on the right apical pulmonary lobe but no distal cancer spread. Additional preoperative positron emission tomography (PET)‐CT with 18F‐fluorodeoxyglucose (18F‐FDG) confirmed the presence of a highly metabolic pulmonary node in the apical portion of the right lung (maximum standardized uptake value 6.3). Thus, the patient underwent thoracic surgery with the right lobectomy technique and associated D2 ilo‐mediastinal lymphadenectomy.

On pathological analysis, macroscopically, the lung lobe measured 13 x 11.5 x 3.5 cm. On the subpleural level there was a grayish nodule with infiltrative margins of 2.5 cm, while the remaining lung tissue was macroscopically normal. The histological exam confirmed moderately differentiated (G2) clear cell adenocarcinoma of the lung with an acinar growth pattern that had infiltrated the lung tissue without visceral pleural involvement. The neoplastic cells had a large and clear vacuolated cytoplasm. The nucleus was round or slightly indented, with finely dispersed chromatin and inconspicuous nucleoli, and several mitotic figures were observed.

**Figure 2:**
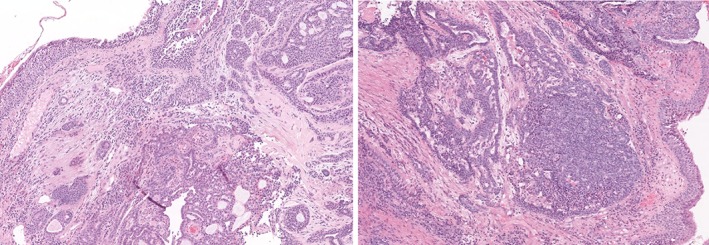
Hematoxylin and eosin stain (x 100) of adenoid cystic carcinoma of the trachea.

**Figure 3:**
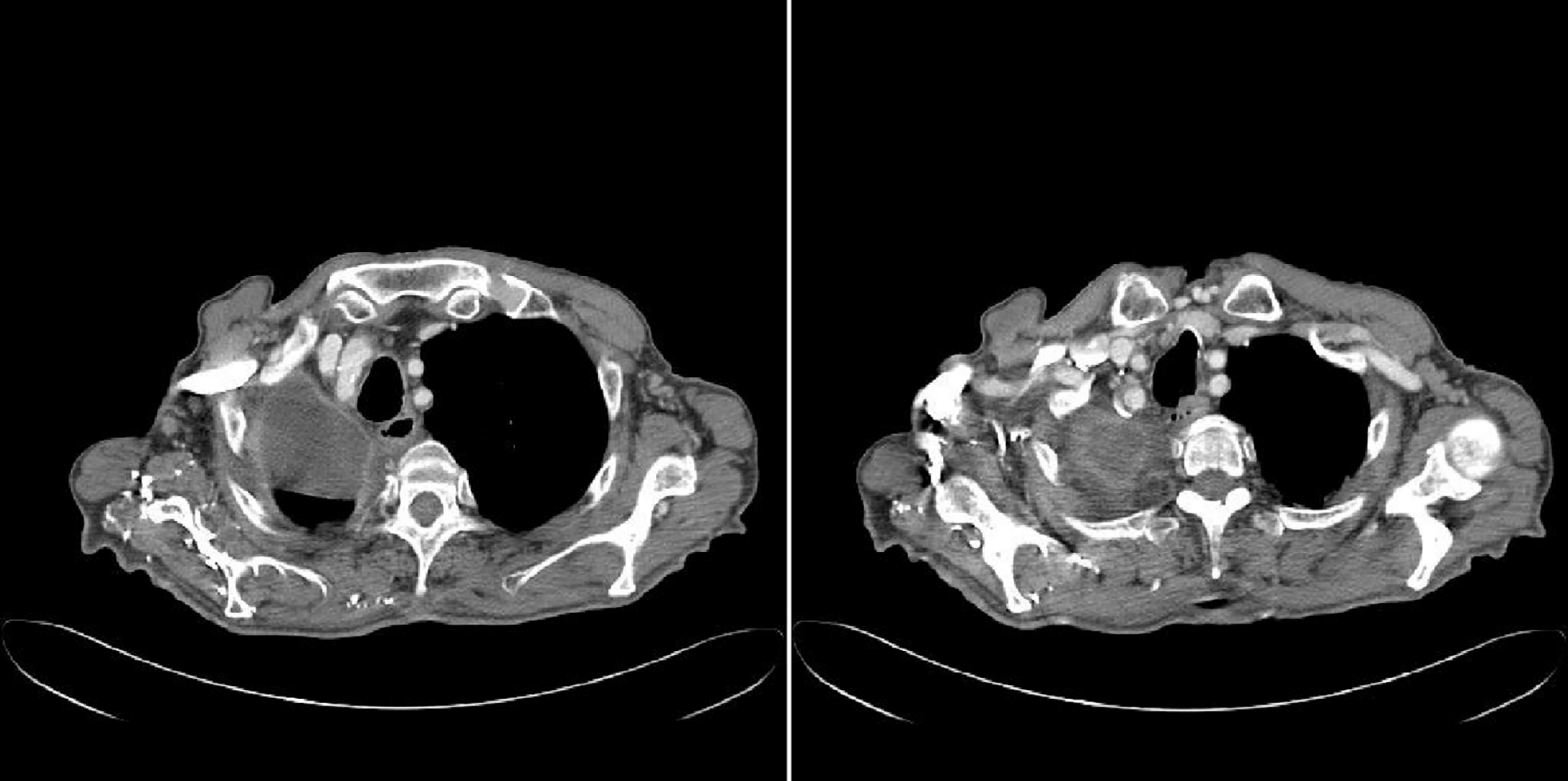
Postoperative total body computed tomography scan, negative for a residual mass.

**Figure 4:**
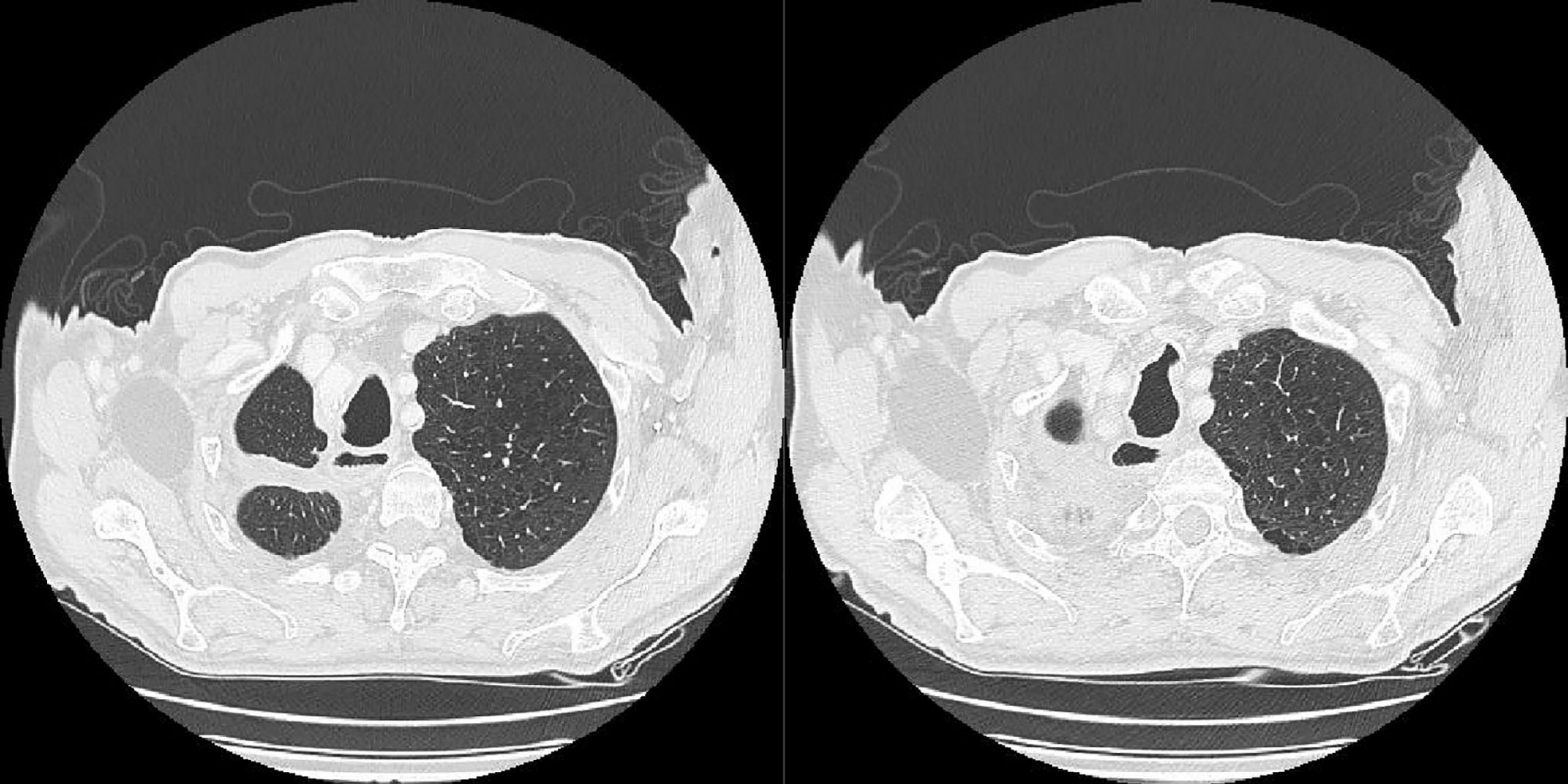
Total body computed tomography scan taken after four years of follow‐up, negative for recurrence or metastatic spread of the primary cancer.

Immunohistological evaluation was positive for CD10, vimentin, pan‐CK, MNF116, and CK7 but negative for TTF‐1 (Fig 2). All 10 of the removed lymph nodes were negative for metastasis. According to current tumor node metastasis stage grouping, the disease was classified as pT1c; pN0 (0/10) cMo (stage IA3). A possible renal origin of the cancer could not be excluded. We performed postoperative radiological evaluation with a total body CT scan that was negative for residual mass, metastatic dissemination, and primary clear cell renal carcinoma (Fig 3). After consultation and considering the absence of any residual illness or metastatic spread, the disease management team decided not to administer adjuvant chemotherapy but to continue specific postoperative oncologic follow‐up. After four years of follow‐up the patient is in good clinical condition with no evidence of recurrent or metastatic disease (Fig 4). Written informed consent was obtained from the patient for publication of this case report and any accompanying images.

## Discussion

A clear cell sugar tumor (CCST), with approximately 60 cases reported in the literature, is an extremely rare mesenchymal tumor that shows varied biological characteristics and clinical presentations. The presence of a clear cell component in pulmonary carcinomas, however, does not constitute a specific clinicopathologic entity; rather, clear cells should be considered a histopathologic feature that may be present in many tumors, including adenocarcinoma, large cell carcinoma, and squamous cell carcinoma. However, the latest WHO classification collocates clear cell carcinomas among primary lung tumors.

Generally asymptomatic and accidentally discovered, most CCSTs are benign. Wang *et al*. reported that CCSTs are usually distributed in the lower lobe of both lungs, unrelated to major bronchi and vessels. Furthermore, they reported that tumor size ranges from 1 mm to 12 cm, showing that tumor size is most closely correlated with symptoms; in particular patients with larger tumors display specific symptoms that could be associated with CCSTs.^4^ Our patient presented with an asymptomatic 22 mm subpleural lesion located in the apical portion of the right lung, suggesting the necessity for surgical treatment.

Contrast‐enhanced CT has been shown to play an important diagnostic role in detecting this type of lung cancer, probably because of its PEC origin, which leads to high vascular growth of capillaries and sinusoidal vessels in its stroma. In fact, intense heterogeneous enhancement in the arterial phase and washout in the delay phase is a common finding. However, occasionally, for unknown reasons, there is no enhancement. In our case report, contrast‐enhanced CT was fundamental for diagnosis and preoperative staging, confirming its essential role in differential diagnosis. CT/18F‐FDG PET is no longer recommended to diagnose renal cell carcinoma because of its high false negative results. This is likely because of the low avidity for glucose in clear cells and their renal excretion, which can mask cancer cells from normal renal tissue. In our case, 18F‐FDG‐PET showed a highly metabolic pulmonary node. CCTLs usually show no 18F‐FDG uptake; however, a benign case with extensive FDG uptake has been reported.^14^

CCTL is usually diagnosed by thoracotomy, lobectomy, and segmentectomy; however, a few cases have been diagnosed with preoperative transbronchial biopsy^15^ or fine‐needle aspiration biopsy.^16^, ^17^ Our patient underwent right lobectomy and lymphadenectomy using the thoracotomy technique for pathological diagnosis.

The biology of CCTLs has traditionally been considered benign; however, certain aggressive cases have been reported, occurring almost exclusively in the lung. One report described the death of a patient from metastatic CCTL,^12^ while two other reports demonstrated rapid growth behavior of this type of lung cancer.^3^, ^13^ The literature suggests that the presence of clear cells does not appear to affect prognosis.^9^, ^18^ Our patient was a heavy smoker with COPD and an associated pulmonary mass, which manifested on CT and PET with highly metabolic behavior. Considering the absence of metastatic spread on radiological examinations, surgical treatment was performed. In the literature, the most common treatments conducted were partial resection and enucleation. Adjuvant therapy has never been considered (Table 1). Our patient underwent right lobectomy and associated D2 ilo‐mediastinal lymphadenectomy, from which no residual tumor mass was detected.

**Table 1:** Type of surgery, adjuvant therapy, and follow‐up in the reviewed CCTL cases

Reference	No. of cases	Patient age (years)	Surgery (no. of patients)	Adjuvant Therapy	Follow‐up
Liebow & Castelman^3^	12	Range 28–64	Lobectomy (5); wedge resection (2); tumor enucleation (3); NA (2)	No	2 years (3), 5 years (1), 7 years (1): alive, no recurrence
Wang *et al*.^4^	1	38	Left thoracotomy and a wedge resection	No	12 months: alive, no recurrence
Papla *et al*.^5^	1	68	NA	NA	NA
Kim *et al*.^10^	1	64	Wedge resection	No	2 months: alive, no recurrence
Hirata *et al*.^11^	1	45	Tumor enucleation	No	16 months: alive, no recurrence
Sale & Kulander^12^	1	NA	Tumor resection	No	Hepatic metastases after 10 years from primary tumor resection
Kalkanis *et al*.^13^	1	46	Wedge resection	No	NA
Zarbis *et al*.^14^	1	71	S6 segmentectomy	No	12 months: alive, no recurrence
Takanami *et al*. 15	1	26	Wedge resection	No	NA
Policarpio‐Nicholas *et al*.^16^	1	64	Wedge resection	No	NA
Edelweiss *et al*.^17^	1	46	Middle lobe segmentectomy	No	6 months: alive, no recurrence
Xu *et al*.^19^	2	24 & 59	Segmentectomy, upper lobectomy	No	12 months and 24 months: alive, no recurrence
Yeon *et al*.^20^	1	58	Wedge resection	No	NA
Tsilimigras *et al*.^21^	1	46	Right middle lobectomy and anterior upper segmentectomy	No	NA

CCTL, clear cell tumor of the lung; NA, not assessed/available.

The principal pathologies to exclude in the differential diagnosis of CCTL are:

- Primary or metastatic adenocarcinoma: generally, it may present as a lobulated mass with an invasive pattern of growth in the surrounding bronchial wall and blood vessels.
- Solitary fibrous tumor: on CT imaging it appears as a mass containing cystic areas, usually originating from the pleura and possibly extending into the pleural cavity.
- Paraganglioma: it can occur as a functional or nonfunctional lesion with different CT contrast enhancement according to the density of blood vessels inside the tumor.
- Pulmonary sclerosing hemangioma: it usually shows a tail or air trapping sign on CT scan.^19^

As clear cell lung carcinoma can also be a manifestation of metastatic renal cell carcinoma or other subtype of clear cell carcinoma originating from different types of lung cells, making a differential diagnosis is a fundamental step before commencing treatment. An important feature that differentiates CCTL from other types of lung cancer, such as metastatic clear cell tumors or other clear cell tumor variants, is the presence of immunoreactivity for CD10, CK, and EMA.^20^, ^21^ CK positivity is a characteristic of both primary and secondary clear cell adenocarcinomas of the lung.^13^ In our case, the diagnosis was based on the typical histological picture of the tumor: positivity for CD10, vimentin, pan‐cytokeratin MNF116, and CK7, and negative for TTF‐1. Renal clear cell carcinoma was excluded because no renal tumor was located on imaging. Moreover, because of its rarity, a revision of the histological examination by an expert pathologist was requested and confirmed the formulated diagnosis.

According to international guidelines, systemic chemotherapy is efficacious for metastases from non‐small cell lung cancer, while adjuvant therapy has never been applied to patients such as ours (Table 1). Considering the absence of any residual illness or metastatic spread, disease management was personalized and was determined by a multidisciplinary team composed of oncologists, surgeons, and radiotherapists who decided to proceed solely with postoperative oncologic follow‐up.

In light of the few cases described in the literature, cytomorphologic aspects, such as growth pattern changes, and clinical features, such as the detection time and observation period, will help clinicians in the diagnostic and therapeutic process, permitting the conservative management of these patients.^20^ Therefore, a better understanding of the pathological, immunophenotype features and natural history of this rare tumor is extremely important to prepare detailed and practical clinical guidelines.

## Disclosure

No authors report any conflict of interest.
